# The culprit insect but not severity of allergic reactions to bee and wasp venom can be determined by molecular diagnosis

**DOI:** 10.1371/journal.pone.0199250

**Published:** 2018-06-25

**Authors:** Pia Gattinger, Christian Lupinek, Lampros Kalogiros, Mira Silar, Mihaela Zidarn, Peter Korosec, Christine Koessler, Natalija Novak, Rudolf Valenta, Irene Mittermann

**Affiliations:** 1 Department of Pathophysiology and Allergy Research, Division of Immunopathology, Center for Pathophysiology, Infectiology and Immunology, Medical University of Vienna, Vienna, Austria; 2 Department of Allergology and Clinical Immunology, 401 General Military Hospital, Athens, Greece; 3 University Clinic of Respiratory and Allergic Diseases, Golnik, Slovenia; 4 Department of Dermatology and Allergy, University of Bonn, Bonn, Germany; Harvard Medical School, UNITED STATES

## Abstract

**Background:**

Allergy to bee and wasp venom can lead to life-threatening systemic reactions. The identification of the culprit species is important for allergen-specific immunotherapy.

**Objectives:**

To determine a panel of recombinant bee and wasp allergens which is suitable for the identification of bee or wasp as culprit allergen sources and to search for molecular surrogates of clinical severity of sting reactions.

**Methods:**

Sera from eighty-seven patients with a detailed documentation of their severity of sting reaction (Mueller grade) and who had been subjected to titrated skin testing with bee and wasp venom were analyzed for bee and wasp-specific IgE levels by ImmunoCAP^TM^. IgE-reactivity testing was performed using a comprehensive panel of recombinant bee and wasp venom allergens (rApi m 1, 2, 3, 4, 5 and 10; rVes v 1 and 5) by ISAC chip technology, ImmunoCAP and ELISA. IgG_4_ antibodies to rApi m 1 and rVes v 5 were determined by ELISA and IgE/IgG_4_ ratios were calculated. Results from skin testing, IgE serology and IgE/IgG_4_ ratios were compared with severity of sting reactions.

**Results:**

The panel of rApi m 1, rApi m 10, rVes v 1 and rVes v 5 allowed identification of the culprit venom in all but two of the 87 patients with good agreement to skin testing. Severities of sting reactions were not associated with results obtained by skin testing, venom-specific IgE levels or molecular diagnosis. Severe sting reactions were observed in patients showing < 1 ISU and < 2kU_A_/L of IgE to Api m 1 and/or Ves v 5.

**Conclusion:**

We identified a minimal panel of recombinant bee and wasp allergens for molecular diagnosis which may permit identification of bee and/or wasp as culprit insect in venom-sensitized subjects. The severity of sting reactions was not associated with parameters obtained by molecular diagnosis.

## Introduction

Systemic allergic reactions to insect venoms affect the skin, the respiratory and gastro-intestinal tract as well as the cardiovascular system and are often life-threatening. According to epidemiologic studies, it is estimated that approximately 1.2–3.5% of the population suffers from severe allergic reactions to insect venom allergens [1–4]. Venoms therefore belong to the most important elicitors of anaphylaxis in children and adults [5, 6]. Insects which most frequently cause severe allergic reactions belong to the order Hymenoptera. The family of Apidae (honeybee, *Apis mellifera*) and Vespidae (wasp; yellow jacket, *Vespula vulgaris*, *Vespula germanica*) and the paper wasp are among the most prevalent ones [7]. Hymenoptera venoms contain a complex mixture of glycosylated and non-glycosylated proteins, peptides and also irritating substances such as enzymes, as well as biogenic amines. Allergen-specific immunotherapy (AIT) is extremely effective for the treatment of venom allergy [8] and shows long-term efficacy [9]. Accurate diagnosis of the culprit insect is therefore important for selection of the right venom for AIT. More than 50% of the patients may show IgE double-positivity to bee and wasp venom [10], which can be due to genuine sensitisation to both venoms, cross-reactivity of IgE with homologous allergens in both venoms or cross-reactivity of IgE with clinically irrelevant carbohydrate epitopes [11]. Evidence has been provided that molecular diagnosis with non-glycosylated recombinant bee and wasp allergens can facilitate the identification of the culprit venom because it eliminates cross-reactive carbohydrate epitopes [11–13]. However, several new bee and wasp allergens have been identified [14, 15]. While their contribution to increase the diagnostic sensitivity of bee and wasp venom allergy has been investigated, much less is known about their value for identifying the culprit sensitizing venom in double-sensitized patients [16–17]. Another important question is whether it is possible to establish surrogate markers similar as in the field of food allergy which allow prediction of the severity of allergic reactions on the basis of allergen-specific sensitivity, allergen-specific IgE levels and/or intensity of IgE recognition of certain marker allergens [18].

Here, we tested a comprehensive panel of recombinant bee and wasp venom allergens for their usefulness to identify the culprit insect in venom allergic patients, which were characterized regarding clinical reactivity by Mueller grading and by skin testing with bee and wasp venom extracts. Furthermore, we investigated if it is possible to establish molecular diagnostics for predicting severities of sting reactions.

## Materials and methods

### Characterization of venom allergic patients and skin testing

Sera from eighty-seven patients with bee and/or wasp venom allergy were analyzed in this study (Tables 1 and 2). For each of the patients a grading of severity of clinical reactions according to Mueller was available (S1 Table). To determine a minimal panel of recombinant bee and wasp allergens for identifying the culprit venom, selected groups of patients were studied: Sixty seven patients of a German population who according to ImmunoCAP testing for specific IgE (sIgE)-reactivity to bee (i1) and wasp extracts (i3) (Thermo Scientific, Phadia AB, Uppsala, Sweden) were double-sensitized to both extracts (G1-G67) (Table 1) and were included as examples of “double positives”. Furthermore, 20 patients from a Slovenian population, 11 were positive only for bee venom (i1) (S1-S11) (Table 2) and 9 were positive only for wasp venom (i3) (S12-S20) were tested as mono-sensitized (Table 2). Serum samples from atopic subjects (n = 40) without a history of hyperreactivity to insect stings were used as control group.

**Table 1:** Summary of serology, skin testing and Mueller grading for the German population.

					Bee							Wasp				
Pat. no.	t-IgE CAP ^a)^	bee CAP ^b)^	wasp CAP ^c)^	Skin test Bee/Wasp ^d)^	rApi m 1 Chip ^e)^ CAP ^f)^		rApi m 2 ELISA ^g)^	rApi m 3 CAP ^h)^	rApi m 4 Chip ^i)^	rApi m 5 CAP ^j)^	rApi m 10 CAP ^k)^	rVes v 5 Chip ^l)^ CAP ^m)^		rVes v 1 ELISA ^n)^	Mueller Grade ^o)^	culprit insect ^p)^
	kU/L	kUA/L	kUA/L	Venom	ISU	kUA/L	O.D.	kUA/L	ISU	kUA/L	kUA/L	ISU	kUA/L	O.D.		
G1	595	1.15	˃100	-1/-2	<0.1	<0.1	0	<0.1	<0.1	<0.1	<0.1	2.27	4.40	0.47	2	w
G2	40.9	6.52	5.11	-1/-1	0.27	<0.1	0.29	<0.1	<0.1	<0.1	<0.1	0.29	0.3	0	2	b/w
G3	148	1.46	4.37	-2/-4	0.29	0.17	0	0.12	<0.1	<0.1	0.28	0.62	0.73	0	0 (LLR)	b/w
G4	223	14.09	2.15	-2/0	1.2	3.6	0.14	n.d.	<0.1	n.d.	0.39	0.24	0.22	0.36	2	b/w
G5	796	1.97	26.9	0/-2	<0.1	<0.1	0	<0.1	<0.1	4.48	<0.1	2.36	3.82	0.42	3	w
G6	252	1.79	8.52	0/-3	<0.1	<0.1	0	0.29	<0.1	0.46	0.93	10.33	1.8	0.62	2	b/w
G7	153	3.03	2.77	-2/-2	0.4	0.17	0	0.14	<0.1	<0.1	<0.1	1.47	2.01	0	2	b/w
G8	65.8	4.43	23.5	0/0	<0.1	<0.1	0	<0.1	<0.1	<0.1	<0.1	2.01	1.14	0	1	w
G9	138	6.41	0.99	-2/0	1.81	1.63	0	n.d.	<0.1	n.d.	2.83	<0.1	<0.1	0	2	b
G10	124	59.9	1.81	-3/-2	10.36	31.4	0	1.72	0.43	0.29	0.16	1.91	1.06	0	1	b/w
G11	12.2	0.51	1.84	-1/-1	<0.1	<0.1	0	<0.1	<0.1	0.62	<0.1	0.96	0.82	0	2	w
G12	189	0.59	25.3	neg/0	<0.1	<0.1	0	<0.1	<0.1	<0.1	<0.1	<0.1	0.13	0.13	2	w
G13	224	5.04	0.71	0/-2	2.96	1.38	0	0.21	<0.1	0.15	0.97	0.48	0.56	0	2	b/w
G14	84.7	7.35	18.3	0/-2	0.49	0.37	0	0.23	<0.1	6.08	0.2	1.19	0.94	0.41	2	b/w
G15	638	50.3	77.5	-1/-2	15.51	8.07	0.19	n.d.	<0.1	n.d.	0.38	7.07	6.23	0.83	3	b/w
G16	435	3.01	9.27	-4/-1	0.7	0.29	0.14	<0.1	<0.1	<0.1	0.56	2.45	4.06	0.56	3	b/w
G17	96	2.2	13.5	neg/-1	<0.1	<0.1	0	<0.1	<0.1	<0.1	<0.1	<0.1	<0.1	0	1	w
G18	61.6	0.46	9.26	neg/-2	<0.1	<0.1	0	<0.1	<0.1	<0.1	<0.1	0.54	2.4	0.14	2	w
G19	171	69.2	17.6	-3/-2	2.75	12.2	0.76	0.18	0.29	1.9	0.84	4.59	4.5	0	3	b/w
G20	61.5	3.8	7.71	-1/-1	0.45	0.73	0	0.24	<0.1	<0.1	0.9	2	3.04	0.32	2	b/w
G21	333	˃100	2.33	-3/-1	1.39	1.89	0.38	1.1	<0.1	0.37	0.4	<0.1	<0.1	0	2	b
G22	123	0.83	5	neg/-2	<0.1	<0.1	0	<0.1	<0.1	<0.1	<0.1	2.32	4.5	0	2	w
G23	451	2.6	10.4	neg/-1	<0.1	<0.1	0	<0.1	<0.1	<0.1	<0.1	0.27	0.53	0	1	w
G24	85.1	0.84	50.7	-1/-1	<0.1	<0.1	0	<0.1	<0.1	0.2	<0.1	1.63	2.39	0.27	1	w
G25	145	31.7	22.4	-2/-1	0.67	0.58	0.32	<0.1	<0.1	<0.1	1.39	1.04	2.02	0.19	3	b/w
G26	7	4.83	3.71	neg/-1	0.21	0.13	0.11	0.12	<0.1	<0.1	<0.1	1.66	0.96	0	2	b/w
G27	324	3.6	91.9	neg/-2	0.42	0.47	0	<0.1	<0.1	0.15	<0.1	2.72	2.46	0.23	2	b/w
G28	59	2.86	26.4	neg/-2	<0.1	<0.1	0	<0.1	<0.1	<0.1	<0.1	1.36	2.67	0.12	1	w
G29	271	0.74	16.5	0/-2	<0.1	<0.1	0	<0.1	<0.1	18.2	<0.1	3.09	3.01	0.18	2	w
G30	238	1.75	62.2	neg/-2	<0.1	<0.1	0	<0.1	<0.1	<0.1	<0.1	0.16	0.29	1.14	1	w
G31	469	˃100	26.1	-3/-3	1.4	2.71	0	0.25	<0.1	<0.1	0.37	0.34	0.66	0.19	2	b/w
G32	26.7	2.83	10.9	-2/-2	<0.1	<0.1	0	<0.1	<0.1	0.4	<0.1	0.61	0.55	0	3	w
G33	43.8	1.43	3.34	-3/-1	0.21	0.39	0.12	<0.1	<0.1	<0.1	0.77	<0.1	<0.1	0.29	1	b/w
G34	78.1	4.1	5.43	-2/-1	0.72	0.62	0	0.14	<0.1	1.03	1.77	1.07	1.14	0.19	2	b/w
G35	146	3.44	9.14	-1/-1	<0.1	0.22	0.11	<0.1	<0.1	2.4	0.76	0.53	1.03	0	1	b/w
G36	157	1.23	1.23	neg/-3	<0.1	<0.1	0	<0.1	<0.1	<0.1	<0.1	0.27	0.52	0.18	2	w
G37	99.2	3.35	5.04	neg/-2	<0.1	<0.1	0	<0.1	<0.1	0.32	<0.1	3.36	2.55	0.47	4	w
G38	645	3.14	14.8	0/-1	<0.1	<0.1	0.21	<0.1	<0.1	0.13	<0.1	1.39	11.2	0.21	2	w
G39	363	8.89	84.8	0/-1	0.86	0.91	0.25	0.14	<0.1	46.8	0.9	22.13	40.4	0.71	2	b/w
G40	78.2	0.36	42.4	-1/-2	0.44	0.15	0	<0.1	<0.1	0.18	<0.1	7.17	6.28	0.11	2	b/w
G41	324	35.8	48.1	neg/0	<0.1	0.18	0.47	<0.1	<0.1	0.15	<0.1	3.51	13.8	0.26	2	w
G42	63.4	3.28	9.75	neg/-1	<0.1	<0.1	0	<0.1	<0.1	<0.1	<0.1	4.46	8.65	0	2	w
G43	158	3.9	9.75	0/-2	<0.1	<0.1	0	<0.1	<0.1	<0.1	<0.1	3.86	6.6	0.14	2	w
G44	63.4	11.6	0.49	-1/neg	2.24	0.47	0	<0.1	<0.1	<0.1	0.35	<0.1	<0.1	0	2	b
G45	654	60.7	10.5	-2/-3	6	12	0.83	0.46	<0.1	7.42	0.19	4.04	21.5	0.16	3	b/w
G46	292	1.71	12	neg/-2	<0.1	<0.1	0	<0.1	<0.1	0.24	<0.1	0.9	1.51	0.28	2	w
G47	460	3.28	9.75	neg/-1	<0.1	<0.1	0	<0.1	<0.1	<0.1	<0.1	1.53	4.57	0	3	w
G48	63.4	0.66	3.88	neg/0	<0.1	0.43	0	0.12	<0.1	0.38	<0.1	0.22	<0.1	0	3	b/w
G49	151	12	78.4	-1/-2	0.27	<0.1	0	0.12	<0.1	0.16	<0.1	1.12	1.97	0.38	3	b/w
G50	566	54.1	31.5	-2/-2	21.93	12.8	0	<0.1	<0.1	<0.1	<0.1	6.39	7.45	0.46	2	b/w
G51	1378	3.55	8.12	0/-2	<0.1	<0.1	0	<0.1	<0.1	<0.1	<0.1	1.41	0.71	0.28	0 (LLR)	w
G52	624	2.94	5.11	0/-1	0.92	1.02	0	0.72	<0.1	<0.1	<0.1	1.34	2.39	0	2	b/w
G53	304	8.81	52.6	-2/-3	3.58	2.37	0	1.08	<0.1	<0.1	<0.1	8.51	10.95	0.22	2	b/w
G54	223	0.45	29.3	0/-1	<0.1	0.29	0	0.21	<0.1	<0.1	<0.1	<0.1	0.36	0	2	b/w
G55	159	2.78	0.89	-4/-1	0.43	0.12	0	0.18	<0.1	3.43	0.67	0.23	0.84	0	2	b/w
G56	474	3.83	1.49	-3/-2	3.9	16.7	0.13	<0.1	<0.1	<0.1	1.47	0.6	0.44	0.35	3	b/w
G57	434	0.55	7.51	neg/-2	<0.1	<0.1	0	<0.1	<0.1	<0.1	<0.1	12.75	18	0.12	2	w
G58	385	79.8	9.07	-3/-3	3.52	2.91	0.11	0.21	<0.1	<0.1	0.6	3.06	5.04	0	2	b/w
G59	162	0.37	27.2	neg/-1	<0.1	<0.1	0	<0.1	<0.1	3.34	<0.1	8.07	7.95	0.16	4	w
G60	562	3.37	11.2	neg/-1	2.15	0.5	0.14	n.d.	<0.1	n.d.	0.36	8.98	6.88	0.4	3	b/w
G61	25.8	30.5	0.6	-2/neg	5.71	6.12	0.39	<0.1	<0.1	0.76	2.87	<0.1	<0.1	0.24	2	b/w
G62	146	0.4	16.6	0/-3	<0.1	<0.1	0	<0.1	<0.1	0.23	<0.1	4.26	1.98	0.53	2	w
G63	50.1	0.64	7.72	0/-2	0.18	<0.1	0	0.2	<0.1	0.4	<0.1	2.24	3.97	0	3	b/w
G64	600	15.6	0.6	-2/neg	1.52	3.34	0.38	0.99	<0.1	0.22	5.7	<0.1	<0.1	0	2	b
G65	3301	4.96	˃100	0/-2	1.93	1.21	0	n.d.	<0.1	n.d.	<0.1	186.8	184.1	0.21	2	b/w
G66	61.5	1.96	3.37	neg/-3	4.43	1.04	0	0.15	<0.1	0.16	0.74	3.37	1.98	0.27	2	b/w
G67	426	63.1	31.9	-2/-3	9.26	9.4	0	n.d.	<0.1	n.d.	<0.1	7.99	5.42	0	4	b/w

a total IgE, ImmunoCAP

b bee venom sIgE, ImmunoCAP

c wasp venom sIgE, ImmunoCAP

d intradermal skin test; given is the threshold concentration of a positive skin reaction, -4: 0.0001 μg/ml, -3: 0.001 μg/ml, -2: 0.01 μg/ml, -1: 0.1 μg/ml, 0: 1 μg/ml venom, neg: no reaction

e IgE-reactivity to non-glycosylated rApi m 1 measured by allergen micro-array

f IgE-reactivity to non-glycosylated rApi m 1 measured by ImmunoCAP

g IgE-reactivity to non-glycosylated rApi m 2 measured by ELISA

h IgE-reactivity to non-glycosylated rApi m 3 measured by ImmunoCAP

i IgE-reactivity to rApi m 4 measured by allergen micro-array

j IgE-reactivity to non-glycosylated rApi m 5 measured by ImmunoCAP

k IgE-reactivity to non-glycosylated rApi m 10 measured by ImmunoCAP

l IgE-reactivity to rVes v 5 measured by allergen micro-array

m IgE-reactivity to rVes v 5 measured by ImmunoCAP

n IgE-reactivity to rVes v 1 measured by ELISA

o clinical severity in Mueller grade or large local reaction

p according to component-resolved diagnosis

Abbr: n.d.: not done, O.D.: optical density, LLR: large local reaction, b: bee, w: wasp, b/w: bee and wasp

IgE-levels ≥ 0.1 ISU, ≥ 0.35 kU_A_/L are highlighted in grey

**Table 2:** Summary of serology, skin testing and Mueller grading for the Slovenian mono-sensitized population.

					Bee							Wasp				
Pat. no.	t-IgE CAP ^a)^	bee CAP ^b)^	wasp CAP ^c)^	Skin test Bee/Wasp ^d)^	rApi m 1 Chip ^e)^ CAP ^f)^		rApi m 2 ELISA ^g)^	rApi m 3 CAP ^h)^	rApi m 4 Chip ^i)^	rApi m 5 CAP ^j)^	rApi m 10 CAP ^k)^	rVes v 5 Chip ^l)^ CAP ^m)^		rVes v 1 ELISA ^n)^	Mueller Grade ^o)^	culprit insect ^p)^
	kU/L	kUA/L	kUA/L	Venom	ISU	kUA/L	O.D.	kUA/L	ISU	kUA/L	kUA/L	ISU	kUA/L	O.D.		
S1	n.d.	2.45	<0.35	10/neg	0.6	1.05	0	<0.1	<0.1	<0.1	<0.1	<0.1	<0.1	0	2	b
S2	17.7	7.82	<0.35	10/neg	0.25	1.59	0	<0.1	<0.1	<0.1	<0.1	<0.1	<0.1	0	2	b
S3	44.8	8.57	<0.35	10/neg	0.49	3.48	0	0.14	0.36	1.9	<0.1	<0.1	<0.1	0	4	b
S4	n.d.	2.85	<0.35	10/neg	0.21	1.31	0.45	n.d.	0.14	n.d.	0.3	<0.1	<0.1	0	3	b
S5	n.d.	1.38	<0.35	100/neg	0.12	0.17	0	<0.1	<0.1	<0.1	<0.1	<0.1	<0.1	0	1	b
S6	n.d.	15.9	<0.35	10/100	1.3	1.59	0.53	<0.1	<0.1	<0.1	<0.1	<0.1	<0.1	0	0 (LLR)	b
S7	212	17	<0.35	10/100	0.83	7.49	0	<0.1	<0.1	6.1	2.53	<0.1	<0.1	0	3	b
S8	n.d.	2.45	<0.35	10/neg	0.6	0.28	0	<0.1	<0.1	n.d.	<0.1	<0.1	<0.1	0	2	b
S9	n.d.	1.69	<0.35	neg/neg	0.29	0.69	0	<0.1	<0.1	<0.1	<0.1	<0.1	<0.1	0	0 (LLR)	b
S10	n.d.	10.7	<0.35	neg/neg	<0.1	<0.1	0.69	<0.1	<0.1	<0.1	<0.1	<0.1	<0.1	0	1	b
S11	57.1	10.9	<0.35	10/neg	0.92	2.63	0	n.d.	0.32	n.d.	16.1	<0.1	<0.1	0	2	b
S12	189	<0.35	2.3	100/100	<0.1	<0.1	0	<0.1	<0.1	<0.1	<0.1	1.11	1.24	0	4	w
S13	224	<0.35	1.96	neg/10	<0.1	<0.1	0	<0.1	<0.1	0.28	<0.1	2.35	1.53	0.58	3	w
S14	84.7	<0.35	11.2	neg/10	<0.1	<0.1	0	<0.1	<0.1	<0.1	<0.1	0.73	0.69	0.29	3	w
S15	638	<0.35	4.67	neg/10	<0.1	<0.1	0	n.d.	<0.1	n.d.	<0.1	1.09	1.43	0.37	2	w
S16	435	<0.35	9.05	neg/100	<0.1	<0.1	0	<0.1	<0.1	<0.1	<0.1	2.38	2.66	0	2	w
S17	96	<0.35	1.94	neg/10	<0.1	<0.1	0	<0.1	<0.1	<0.1	<0.1	0.67	0.58	0.28	3	w
S18	61.6	<0.35	3.57	neg/10	<0.1	<0.1	0	<0.1	<0.1	<0.1	<0.1	2.39	2.02	0.41	4	w
S19	171	<0.35	8.19	neg/100	<0.1	<0.1	0	<0.1	<0.1	0.43	<0.1	3.44	3.16	0	2	w
S20	61.5	<0.35	4.93	neg/10	<0.1	<0.1	0	<0.1	<0.1	<0.1	<0.1	3.41	5.29	0	4	w

a) total IgE, ImmunoCAP

b) bee venom sIgE, ImmunoCAP

c) wasp venom sIgE, ImmunoCAP

d) skin prick test; given is the threshold concentration of a positive skin reaction

10 μg/ml, 100 μg/ml, neg: no reaction

e) IgE-reactivity to non-glycosylated rApi m 1 measured by allergen micro-array

f) IgE-reactivity to non-glycosylated rApi m 1 measured by ImmunoCAP

g) IgE-reactivity to non-glycosylated rApi m 2 measured by ELISA

h) IgE-reactivity to non-glycosylated rApi m 3 measured by ImmunoCAP

i) IgE-reactivity to rApi m 4 measured by allergen micro-array

j) IgE-reactivity to non-glycosylated rApi m 5 measured by ImmunoCAP

k) IgE-reactivity to non-glycosylated rApi m 10 measured by ImmunoCAP

l) IgE-reactivity to rVes v 5 measured by allergen micro-array

m) IgE-reactivity to rVes v 5 measured by ImmunoCAP

n) IgE-reactivity to rVes v 1 measured by ELISA

o) clinical severity in Mueller grade or large local reaction

p) according to component-resolved diagnosis

Abbr: n.d.: not done, O.D.: optical density, LLR: large local reaction, b: bee, w: wasp

IgE-levels ≥ 0.1 ISU, ≥ 0.35 kU_A_/L are highlighted in grey

The Slovenian mono-sensitized patients had been tested by skin prick testing with two concentrations of bee and wasp venom extracts (i.e., 10 and/or 100 μg/mL) (HAL Allergy, Leiden, Netherlands). The skin prick test results were analyzed as described [19]. For the German patients titrated intradermal skin testing was performed with 0.02 ml of an initial concentration of 0.0001 μg/ml of bee and wasp venom extract (Alk-Abello Arzneimittel GmbH, Hamburg, Germany) [20]. For each successive test the venom concentration was increased 10-fold (i.e., 0.001 μg/ml; 0.01 μg/ml; 0.1 μg/ml) until a positive reaction was elicited or a maximum concentration of 1 μg/ml (dilution 0) was reached (Table 1). Histamine was used as positive control and NaCl as negative control (Allergopharma GmbH &Co. KG, Reinbek, Germany). Skin test titrations were analyzed according to Turkeltaub *et al* [21]. None of the patients had received venom immunotherapy before. The anonymized analysis of the sera was approved by the ethical committee of the Medical University of Vienna (EK1641/2014).

### Recombinant allergens, protein expression, purification

Synthetic codon-optimized genes coding for Ves v 1 or Ves v 5 were inserted into plasmid pET17b and expressed in *Escherichia coli* BL21 (DE3) as C-terminally his-tagged proteins and purified by inclusion body preparation and Ni^2+-^chelate affinity chromatography [12]. Recombinant (r) Api m 1 and rApi m 2 were expressed in insect-cells as his-tagged protein and purified by Nickel-affinity chromatography. *Spodoptera frugiperda* (Sf9) insect cells were used as hosts for the baculovirus vector [22]. In the cDNA coding for Api m 1 the single N-glycosylation site (N-x-S/T) (Asparagine-x (any amino acid)-Serine/Threonine) and for Api m 2 both N-glycosylation sites were mutated by an exchange of Asparagine to Glutamine, so that the recombinant proteins were not glycosylated. Protein concentrations were determined by Micro BCA Protein Assay (Pierce, Rockford, IL). Purity of the proteins was checked by SDS-PAGE and Coomassie Blue staining [23]. A protein molecular weight marker (PageRuler prestained Protein Ladder Plus, Fermentas, St Leon-Rot, Germany) was used as a standard.

### Measurement of allergen-specific IgE and IgG_4_ antibodies

Specific IgE antibodies of serum samples from bee and/or wasp venom allergic patients were quantified by ImmunoCAP tests containing the recombinant (r) allergens rApi m 1 (i208), rApi m 3 (i215), rApi m 5 (i216), rApi m 10 (i217) and rVes v 5 (i209) (Thermo Fisher, Phadia AB). Specific IgE values ≥ 0.35 kU_A_/L were considered positive and are highlighted in grey in Tables 1 and 2. Sera from atopic patients without a history of hyperreactivity to insect stings were assessed by ImmunoCAP (Thermo Fisher, Phadia AB) for IgE specific for rApi m 1 and rVes v 5 (S2 Table). Additionally, total IgE levels were determined in all sera by ImmunoCAP and for certain sera carbohydrate-specific IgE was assessed using MUXF3 (o214), horseradish peroxidase (HRP) and ascorbate oxidase (ASOD) ImmunoCAP tests.

For all sera IgE-reactivities to more than 150 micro-arrayed allergen components were analyzed with a customized allergen-chip based on the ISAC technology (Thermo Fisher, Phadia) as described [24]. After washing and drying the chips, 35μl aliquots of undiluted serum samples were applied and incubated for 2h at gentle rocking. After another washing, 35μl of an anti-IgE antibody (Phadia AB) labelled with a fluorochrome was applied and incubated for 30min. Washed and dried arrays were analyzed by using a confocal laser scanner (LuxScan-10 K microarray scanner, Capital-Bio, Beijing, People’s Republic China) and evaluated by Microarray Image Analyzer v3.1.2 software (Phadia AB, Uppsala, Sweden). For calibration and detection of background signals a calibrator serum and sample diluent (Phadia AB) were included in each analysis. The results were given as ISAC standardized units (ISU) and values ≥0.1 ISU were considered positive.

IgE reactivity to purified rApi m 2 expressed in insect cells as folded and non-glycosylated protein and to *E*. *coli*-expressed rVes v 1 was studied by IgE ELISA. Patient`s sera were diluted 1:5 in PBS containing 0.5% wt/vol BSA and 0.05% vol/vol Tween 20 and tested for IgE-reactivity to the allergens and, for control purposes, to human serum albumin (HSA). Bound IgE was detected as described [25]. IgG_4_ antibody levels specific for rApi m 1 and rVes v 5 were measured in 1:40 diluted serum samples by ELISA as described [26]. IgE(kU_A_/L)/IgG_4_(OD) ratios for Ves v 5 were calculated as described [27].

### Statistical analyses

Correlation between levels of allergen specific IgE measured by allergen chip or ImmunoCAP was analyzed according to Pearson. The same test was also used to analyze the possible correlation of skin test results or allergen specific IgE-levels with the severity of the sting reaction. Results with a p-value <0.05 were considered significant.

## Results

### Molecular diagnosis allows the identification of the culprit venom responsible for sting reactions

**Fig 1:**
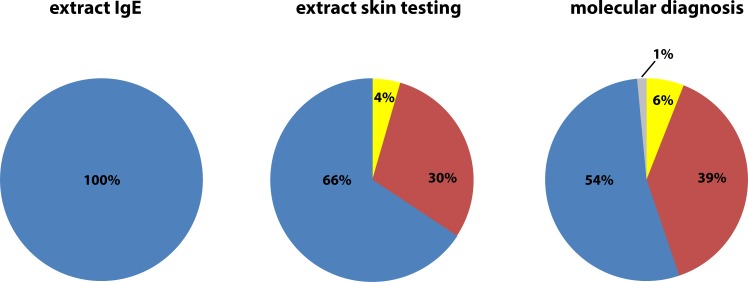
Bee and wasp venom sensitization according to allergen-extract-based serology, skin testing and molecular diagnosis. Pie charts showing the percentages of patients with bee and wasp venom double sensitization (blue), sensitization to bee (yellow) and wasp (red) according to allergen extract-based serology (left), skin testing (middle) and molecular diagnosis (right; one subject negative: grey) in the German population (n = 67).

Tables 1 and 2 provide an overview of the study populations. We investigated 67 patients from Germany (G1-G67, Table 1), which have been selected for double positive sIgE reactivity to bee and wasp venom extract in ImmunoCAP measurements (Table 1) (Fig 1, extract IgE). Out of 20 mono-sensitized patients from Slovenia (S1-S20, Table 2), 11 were IgE positive only to bee but not to wasp venom and 9 were positive only to wasp, but not bee venom (Table 2) (S1 Fig). Patients from both populations had experienced allergic sting reactions which had been graded according to Mueller (S1 Table). Five % of the patients had experienced a large local reaction (LLR) and 95% had shown a systemic allergic reaction. The severities of the systemic reactions in the patients (n = 87) were as follows: Mueller grade 1: 13%, grade 2: 54%, grade 3: 21%, grade 4: 8% (S1 Table).

In a control group (n = 40) of atopic patients without hyperreactivity reactions to insect stings, 28% showed IgE-reactivity to bee venom and 35% to wasp venom in ImmunoCAP measurements (≥0.35kU_A_/L) (S2 Table).

Venom allergic German as well as Slovenian patients were also subjected to skin testing with bee and wasp venom extract. Sixty six % of the German patients, who were thought to be double-sensitized according to allergen extract-based IgE serology, were found to be double-sensitized by skin testing whereas 4% and 30% were identified as mono-sensitized to bee and wasp, respectively (Fig 1, extract skin testing, Table 1). Molecular diagnosis based on a panel of recombinant bee and wasp venom allergens including the established marker allergens for bee and wasp sensitization (i.e., Api m 1: bee; Ves v 1 and Ves v 5: wasp) and the more recently described bee venom allergens Api m 3, 5 and 10 showed that 54% were double-sensitized and 6% and 39% were mono-sensitized to bee and wasp, respectively (Fig 1, Table 1). rApi m 10 helped to identify 2 Api m 1-negative patients (i.e., patient G6 and G35, Table 1) as being bee-sensitized. All rApi m 3 and rApi m 4 positive patients showed also IgE reactivity to rApi m 1. rApi m 5 which cross-reacts with the wasp allergen Ves v 3 was not helpful for the diagnosis of a genuine bee sensitization. In fact, IgE reactivity to rApi m 5 was observed in 5 patients without IgE reactivity to other bee-specific allergens (i.e., rApi m 1, rApi m 3, rApi m 4 or rApi m 10) who reacted with Ves v 5 and therefore are most likely wasp-sensitized. Twelve other patients who showed IgE reactivity to rApi m 5 were sensitized both to rApi m 1 and Ves v 5 and therefore were classified as double-sensitized to bee and wasp. Only one patient was not detected by molecular diagnosis (G17, Table 1, Fig 1: grey).

None of the sera from the control group, without hyperreactivity reactions to bee and/or wasp venom, showed IgE-reactivity to rApi m 1, but 22.5% and 15% showed IgE reactivity to rVes v 5 by allergen chip and ImmunoCAP measurements, respectively (S2 Table).

Skin testing of the Slovenian patients yielded negative results for 18% of the patients (grey part, S1 Fig) who, according to serology, were classified as bee-sensitized (S9, S10, Table 2), whereas 18% showed positive skin reactions to both venoms (blue part S1 Fig; S6, S7, Table 2). All of the wasp venom allergic patients from Slovenia displayed skin test sensitivity to wasp venom, one patient (11%) showed also a positive skin reaction with 100 μg/mL bee venom (S12) (Table 2, S1 Fig). However, molecular diagnosis with rApi m 1, rApi m 2 and rVes v 5 confirmed mono-sensitization to bee and wasp in all of the Slovenian patients (S1 Fig, Table 2). One of the bee venom mono-sensitized patients (S10) showed IgE-reactivity to rApi m 2, without IgE-reactivity to any of the bee venom specific allergens tested (Table 2).

We also investigated IgE sensitization to carbohydrate-containing plant allergens and markers for carbohydrate-sensitization in the German and Slovenian population. Fifty-four % of the bee and wasp venom double-sensitized German patients and 20% of the mono-sensitized Slovenian patients reacted with the natural glycosylated timothy grass pollen allergen nPhl p 4 and/or the glycosylated Bermuda grass pollen allergen nCyn d 1, without IgE reactivity to the major marker allergens for grass pollen sensitization, rPhl p 1, rPhl p 5 or rPhlp 6 (S3 and S4 Tables). From the German population, 10 sera (15%) showed IgE-reactivity to bee venom extract, without any reactivity to rApi m 1, rApi m 2, rApi m 3, rApi m 4, rApi m 5 and rApi m 10 but with IgE-reactivity to nPhl p 4, suggesting that serological positivity to bee venom was due to carbohydrate-specific IgE cross-reactivity. Skin testing of these 10 patients with bee venom showed that 5 patients were negative, whereas the other 5 patients showed positive results only with highly concentrated venom (3 subjects with 1 μg/ml and 2 patients with 0.1 μg/ml of bee venom).

### Skin sensitivity to allergen extracts is not correlated with severity of allergic sting reactions

**Fig 2:**
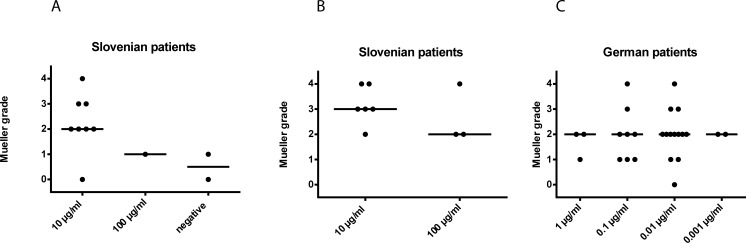
Association of skin sensitivity and severity of sting reaction. Large local reaction (0) and Mueller grade (1–4) of sting reactions (y-axes, medians: horizontal lines) to (A) bee and (B), (C) wasp are shown for Slovenian and German patients with identified culprit venom, and were plotted against the lowest concentration (x-axes) causing a positive reaction in skin prick (Slovenian patients) or intradermal testing (German patients).

Next we investigated if skin sensitivity and severity of allergic sting reactions are correlated. Fig 2A shows the skin sensitivity of the bee venom allergic patients in relation to the severity of sting reactions (0: LLR; Mueller grading 1–4). The number of bee-sensitized patients was low (n = 11) and a statistical analysis was therefore not performed (Fig 2A). However, the results showed that there was a large variation in clinical sensitivity even in patients who showed a skin reaction to the lowest concentration. Local reactions but also grade 4 reactions were found for this threshold (Fig 2A). Similar results were found for wasp venom sensitized patients. There was no association between the extent of skin sensitivity and the severity of sting reactions neither in the Slovenian patients who had been skin prick tested (Fig 2B) nor in the German population for whom intradermal testing was performed (Fig 2C).

### Allergen extract-specific IgE levels are not correlated with severity of allergic sting reactions

**Fig 3:**
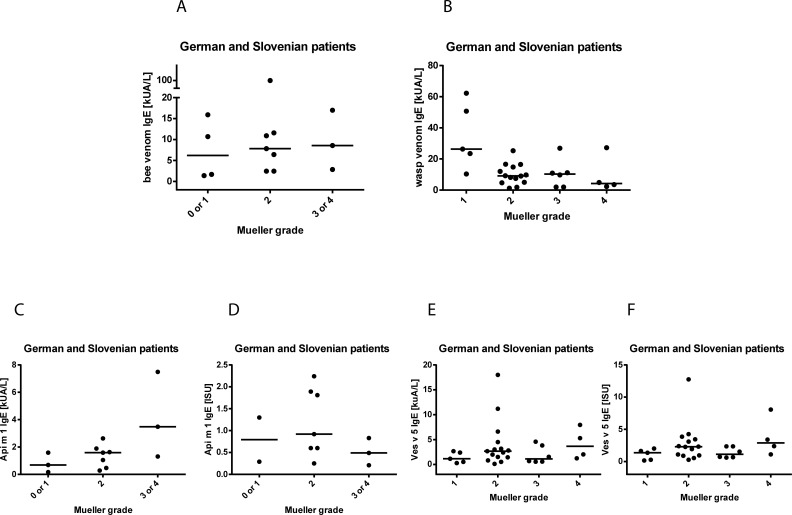
Association of venom extract or allergen-specific IgE levels and severity of sting reactions. Specific IgE levels (medians: horizontal lines) to (A) bee and (B) wasp venom (kU_A_/L), (C) rApi m 1 (kU_A_/L), (D) rApi m 1 (ISU), (E) rVes v 5 (kU_A_/L), (F) rVes v 5 (ISU) were plotted against the severities of sting reactions (x-axes: Mueller grade) for Slovenian and German patients with identified culprit insect.

Fig 3A shows the bee venom-specific IgE levels determined for the bee venom allergic patients from Slovenia and Germany in relation to severity of sting reactions. There were no differences regarding the levels of specific IgE in patients with mild or severe symptoms (Fig 3A). Similar results were found for the wasp venom allergic patients from Germany and Slovenia (Fig 3B). Again there was no association between the venom-specific IgE levels and the severity of sting reactions to bee or wasp. For example, patients had wasp-specific IgE levels of 8–62.2 kU_A_/L but only large local or grade 1 reactions (G8, G23, G24, G28, G30, G51, Table 1), and a similar distribution of wasp-specific IgE levels (i.e., 5–27 kU_A_/L) was found for patients with severe reactions (e.g., G5, G32, G37, G47, G59, Fig 3B, Tables 1 and 2).

### IgE reactivity to recombinant marker allergens for bee and wasp venom sensitization is not correlated with severity of allergic sting reactions

Since natural bee and wasp venom extracts contain non-allergenic but IgE-reactive carbohydrate epitopes and a considerable number of the German patients had shown carbohydrate specific IgE reactivity (S3 Table), we used the non-glycosylated marker allergens rApi m 1 (bee) and rVes v 5 (wasp) to investigate if marker allergen-specific IgE levels are associated with severity of sting reactions (Fig 3C–3F). We used two different technologies for measuring allergen-specific IgE levels. Quantitative ImmunoCAP measurements performed in allergen excess as well as the ISAC chip technology, measuring allergen-specific IgE towards low amounts of solid phase bound allergen, which mimic more closely *in vivo* conditions because blocking IgG antibodies can compete with IgE and reduce the IgE signal [24, 28, 29]. When we compared both technologies we found that 55% of the sera showed IgE reactivity ≥0.1 ISU to rApi m 1 by chip measurements compared to 43% (cutoff ≥ 0.35 kU_A_/L) as detected by ImmunoCAP. Using a cutoff of ≥ 0.1 kU_A_/L for the ImmunoCAP measurements, 57% of the sera showed IgE-reactivity to rApi m 1. There was a significant correlation between IgE levels determined with both methods (R: 0.686, p<0.0001) (S2A Fig).

A similar correlation was found for rVes v 5-specific IgE reactivities. We found that 87% of the patients showed IgE-reactivity to rVes v 5 ≥ 0.1 ISU by chip technology and 82% of the sera were positive for rVes v 5 by ImmunoCAP measurements (cutoff ≥ 0.35 kU_A_/L; 88% positive using cutoff ≥ 0.1 kU_A_/L). Again the levels of Ves v 5-specific IgE determined by both technologies were significantly correlated (R: 0.986, p<0.0001) (S2B Fig).

Since Api m 1 and Ves v 5 are the marker allergens for bee and wasp sensitization, we investigated if there is an association between skin sensitivity and allergen-specific IgE levels to these allergens (S3 Fig). However, there was no clear association between Api m 1-specific IgE levels determined by quantitative ImmunoCAP measurements (S3A Fig) or by allergen-chip (S3B Fig). We only noted that IgE levels were somewhat higher in the patients who reacted already at concentrations of 0.01 μg/ml and 0.001 μg/ml but those patients with the highest skin sensitivity (i.e., 0.0001 μg/ml) had low allergen-specific IgE levels. Similar findings were made for Ves v 5, where there was no association between skin sensitivity to wasp extract and Ves v 5 IgE levels determined by ImmunoCAP (S3C Fig) or chip (S3D Fig).

**Fig 4:**
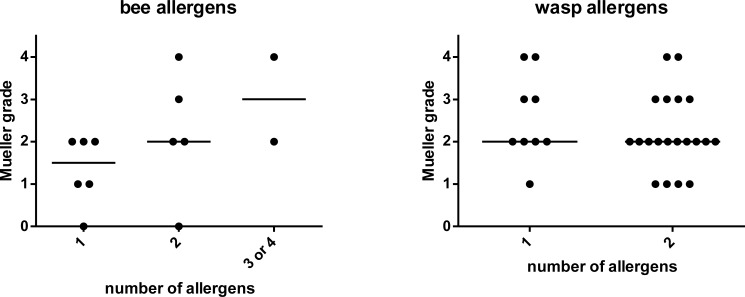
Relation between the number of recognized allergen molecules and severity of sting reactions. The severity of sting reactions (y-axes) is plotted against the number of recognized bee (A) or wasp allergens (B) in the bee or wasp-sensitized patients. Horizontal lines denote medians.

Fig 3C and 3D show that there was also no relation between rApi m 1-specific IgE levels measured by ImmunoCAP (Fig 3C) or allergen chip (Fig 4D) and the severity of sting reactions in bee venom allergic patients from Germany and Slovenia. There was no relevant difference regarding rApi m 1-specific IgE levels between subjects with mild or severe reactions (Fig 3C and 3D). Importantly, also subjects with low IgE sensitization (i.e., <2 kU_A_/L, <1 ISU) to Api m 1 without IgE reactivity to Api m 3, 4, 5 or 10 showed quite severe sting reactions (i.e., Mueller grade 2) (Table 2: patients S1, S2, S8).

Similar results were obtained for rVes v 5 (Fig 3E and 3F). There were no relevant differences between patients with mild or severe sting reactions and the levels of Ves v 5-specific IgE determined by ImmunoCAP (Fig 3E) or chip technology (Fig 3F). Also for rVes v 5 we found mono-sensitized subjects with low IgE levels to rVes v 5 (i.e., <2 kU_A_/L, < 1 ISU) who had experienced severe sting reactions (i.e., Mueller grade 3) (Table 2: patients S14, S17).

Furthermore we measured IgG_4_ levels to rApi m 1 and rVes v 5 for patients with identified culprit insect and available Mueller grading (Fig 3C and 3E). Relevant Api m 1-specific IgG_4_ levels (OD ≥0.1) were detected only in 5 out 13 sera and for Ves v 5 in 20 out of 30 sera. IgE/IgG_4_ ratios were therefore calculated only for Ves v 5. For these patients no association between the IgE/IgG_4_ ratio and the severity of sting reactions was found (S4 Fig).

In addition, we analysed the number of recognized bee and wasp allergens in relation to the severity of sting reactions (Fig 4). Sera from bee venom allergic patients with Mueller grade 3 or 4 symptoms recognized at least 2 bee venom allergens. However grade 2 reactions were observed in subjects recognizing one, two or three-four allergens (Fig 4A).There was no difference regarding the severity of sting reactions and the number of recognized wasp allergens (Fig 4B).

## Discussion

**Fig 5:**
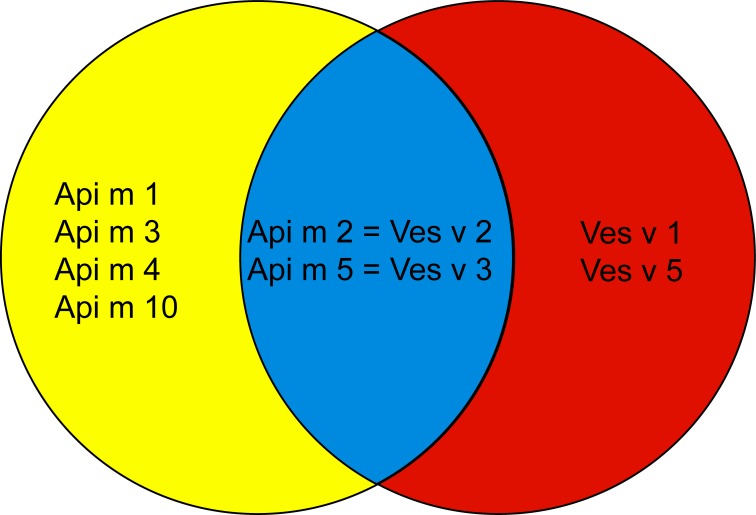
Schematic representation of recombinant marker allergens for diagnosis of genuine IgE sensitization to bee (Api m 1, 3, 4 and 10) and wasp (Ves v 1 and 5) and cross-reactive marker allergens (Api m 2 = Ves v 2; Api m 5 = Ves v 3).

In this study we have addressed two aspects in the diagnosis of allergy to bee and wasp venom. The first aspect concerns the identification of the culprit insect for prescription of AIT. In fact, AIT of bee and wasp venom allergy is highly effective, but it is often very difficult to determine the culprit venom responsible for the allergic reaction in order to prescribe the correct venom for AIT. It has been shown that “false” positive test results can be obtained by allergen-extract based IgE serology due to the presence of clinically irrelevant cross-reactive carbohydrate epitopes in natural allergen extracts [10, 12, 30, 31]. Furthermore, it is known, that venom extracts contain toxic, pharmacologically active and irritating substances which may cause false positive skin test results [32]. The use of pure recombinant allergen molecules from bee and wasp, which can be produced in a non-glycosylated form for IgE-serology, has been suggested as a possibility to discriminate between IgE sensitizations to bee and wasp [10–12]. The initial studies suggested that rApi m 1 from bee and rVes v 5 from wasp are suitable marker allergens to identify bee and wasp sensitized patients, respectively. However, several additional new bee and wasp allergens have then been characterized [14–16] and the question remained what panel of allergen molecules may be required for the discrimination between bee and wasp sensitization. Fig 5 displays a scheme of the currently available marker allergens for identifying genuine IgE sensitizations to bee (i.e., Api m 1, 3, 4 and 10) and wasp (i.e., Ves v 1 and 5) as well as of cross-reactive marker allergens (i.e., Api m 2 = Ves v 2; Api m 5 = Ves v 3). In this study we used rApi m 1, 2, 3, 4, 5 and 10 and Ves v 1 and 5 for IgE-based serology.

Our results obtained in the 67 subjects who were double-positive to bee and wasp venom extract indicate that the panel of rApi m 1 and 10 as well as of rVes v 1 and 5 may be sufficient to identify the culprit sensitizing venom. Testing with the panel of recombinant non-glycosylated allergens especially avoided false positive IgE reactivity results due to cross-reactive carbohydrate epitopes in patients containing carbohydrate-specific IgE (S3 Table). Moreover molecular diagnosis showed that in some cases skin test double positivity to bee and wasp venom was due to IgE reactivity to cross-reactive allergens (e.g., Api m 2, Api m 5) and was also superior to skin testing which gave rise to false negative and false positive test results.

In the patients tested by us, Api m 3 and Api m 4 were not required in addition to Api m 1 and 10 to identify bee-sensitized patients. Due to IgE cross-reactivity with Ves v 3, Api m 5 was not useful for the identification of bee-sensitized patients. Testing with rApi m 2 which cross-reacts with Ves v 2 identified one out of eleven bee venom mono-sensitized patients who showed neither a positive reaction in skin prick tests using bee and wasp venom nor any of the other bee or wasp allergens tested. However, no additional information was obtained by testing with Api m 2 for the other 86 tested patients.

Due to the fact that for all of the investigated patients detailed data regarding the severity of sting reactions according to Mueller grading and the intensity of skin sensitivity through titrated skin tests were available, we could investigate if surrogate markers can be defined which would allow to predict the severity of sting reactions. This question is very important, because the severity of a reaction varies, ranging from mild local to severe and life-threatening anaphylaxis. Unlike as for food allergy, where it is possible to define threshold levels of allergen-specific IgE and/or IgE reactivity profiles to certain high risk allergen molecules [33–35], that are indicative of severe allergic reactions, we were not able to establish associations between the severity of the sting reaction and quantitative allergen-extract-specific IgE levels, which is in agreement with earlier studies [36, 37]. However, even with the use of highly pure and clinically relevant non-glycosylated bee and wasp allergen molecules we could not identify serological surrogate parameters for severity of sting reactions. In fact, we measured allergen-specific IgE levels by two technologies, one highly sensitive test measuring specific IgE in allergen excess (i.e., ImmunoCAP) and another one measuring IgE under conditions of low allergen concentration which allows competition of IgG with IgE and thus mimicking more closely *in vivo* conditions (i.e., ISAC chip technology) [28]. Results obtained with both technologies for allergen-specific IgE were highly correlated but did not allow to predict the severity of sting reactions [38]. In addition we investigated the possible value of the IgE/IgG_4_ ratio specific for Ves v 5 for predicting clinical severity in wasp-sensitized patients but also this parameter was not associated with Mueller grading. Moreover, a considerable percentage of individuals without hyperreactivity reactions to insect venoms showed IgE reactivity to bee and wasp venom extracts and to rVes v 5 indicating that serological IgE test results have no predictive value for clinical severity in bee and wasp sensitization.

Importantly, we found patients who had very low levels of specific IgE against the clinically relevant marker allergens from bee (rApi m 1) and wasp (rVes v 5) but exhibited very severe sting reactions. We consider this result to be of high clinical relevance because it indicates that even subjects with very low IgE levels against these marker allergens can experience severe reactions. Accordingly one may consider informing patients with a positive IgE test results to the non-glycosylated marker allergens (Api m 1, Ves v 5) about the potential risk that they may experience a severe sting reaction.

## Supporting information

S1 Fig Bee and wasp sensitization according to allergen-extract-based serology, skin testing and molecular diagnosis. Pie charts showing the percentages of patients with bee and wasp double sensitization (blue), sensitization to bee (yellow), wasp (red) and without positive skin prick test result (grey) according to allergen extract-based serology (left), skin testing (middle) and molecular diagnosis (right) in Slovenian patients with mono-sensitization to (A) bee (n = 11) and (B) wasp (n = 9).
(EPS)

S2 Fig **Correlation of marker allergen-specific sIgE levels to (A) rApi m 1 and (B) rVes v 5 determined by quantitative ImmunoCAP (y-axes: kU**_**A**_**/l) and ISAC allergen-chip measurements (x-axes: ISU) as scatter plots.** Pearson`s correlation coefficients for rApi m 1: R = 0.686, p<0.0001and for rVes v 5: R = 0.986, p<0.0001.
(EPS)

S3 Fig Association between specific IgE levels to marker allergens and skin test sensitivity. Specific IgE levels to Api m 1 or Ves v 5 measured by (A), (C) ImmunoCAP (kU_A_/L) or (B), (D) allergen chip (ISU) (y-axes) were plotted against the lowest concentration of venom giving a positive reaction in intradermal skin testing (x-axes).
(EPS)

S4 Fig Association between the IgE/IgG_4_ ratios of Ves v 5 and the severity of the sting reactions. Ratios of Ves v 5 specific IgE/IgG_4_ (y-axis) were plotted against the severities of sting reactions (x-axes) for Slovenian and German patients with identified culprit insect.
(EPS)

S1 Table Classification of bee and/or wasp venom allergic patients from Germany and Slovenia according to Mueller. (DOCX)

S2 Table IgE-reactivity to bee and wasp venom allergen extracts and to the major allergens Api m 1 and Ves v 5 in a control population of atopic subjects without history of hyperreactivity to insect stings. (DOCX)

S3 Table IgE–reactivity to marker allergens for carbohydrate sensitization and to the major timothy grass pollen allergens in the German population. (DOCX)

S4 Table IgE-reactivity to marker allergens for carbohydrate sensitization and to the major timothy grass pollen allergens in mono-sensitized Slovenian population. (DOCX)
