# Supplementary material for: The culprit insect but not severity of allergic reactions to bee and wasp venom can be determined by molecular diagnosis
Source: PLoS One. 2018 Jun 25;13(6):e0199250. doi: 10.1371/journal.pone.0199250 (PMC6016944; doi:10.1371/journal.pone.0199250)
Supplement: S1 Table — (DOCX) [file pone.0199250.s005.docx]

**S1 Table: Classification of bee and/or wasp venom allergic patients from Germany and Slovenia according to Mueller**

| Classification | Symptom | n=87  positive n (%) |
| --- | --- | --- |
| LLR | Large local reaction  Swelling exceeding a diameter of 10 cm for at least 24 h | 4 (5) |
| Mueller grade 1 | Large local reaction, pruritus, urticaria, facial swelling, heat, conjunctivitis | 11 (13) |
| Mueller grade 2 | Any grade 1 reaction, angioedema, tightness in the chest, nausea, vomiting, dizziness | 47 (54) |
| Mueller grade 3 | Any grade 1 + 2 reaction, hoarseness, dyspnea, hypotension, weakness | 18 (21) |
| Mueller grade 4 | Any grade 1, 2 + 3 reaction, incontinence, collapse, loss of consciousness, cyanosis | 7 (8) |
