# Supplementary material for: The culprit insect but not severity of allergic reactions to bee and wasp venom can be determined by molecular diagnosis
Source: PLoS One. 2018 Jun 25;13(6):e0199250. doi: 10.1371/journal.pone.0199250 (PMC6016944; doi:10.1371/journal.pone.0199250)
Supplement: S2 Table — (DOCX) [file pone.0199250.s006.docx]

**S2 Table. IgE-reactivity to bee and wasp venom allergen extracts and to the major allergens Api m 1 and Ves v 5 in a control population of atopic subjects without history of hyperreactivity to insect stings**

| no. | bee  CAP ^a)^ | wasp  CAP ^b)^ | rApi m 1  Chip ^c)^ CAP ^d)^ | | rVes v 5  Chip ^e)^ CAP ^f)^ | |
| --- | --- | --- | --- | --- | --- | --- |
|  | kU_A_/L | kU_A_/L | ISU | kU_A_/L | ISU | kU_A_/L |
| C1 | 0.13 | 0.04 | <0.1 | <0.1 | 0.35 | 0.14 |
| C2 | 16.1 | 0.20 | <0.1 | 0.16 | <0.1 | <0.1 |
| C3 | 2.82 | 8.46 | <0.1 | <0.1 | <0.1 | <0.1 |
| C4 | 0.54 | 0.94 | <0.1 | <0.1 | <0.1 | <0.1 |
| C5 | 16.9 | 6.03 | <0.1 | <0.1 | 0.22 | 0.37 |
| C6 | 0.83 | 1.75 | <0.1 | <0.1 | <0.1 | <0.1 |
| C7 | 17 | 6.82 | <0.1 | <0.1 | 0.57 | 0.60 |
| C8 | 0.61 | 0.41 | <0.1 | <0.1 | <0.1 | <0.1 |
| C9 | 0.41 | 7.49 | <0.1 | <0.1 | 10.16 | 7.13 |
| C10 | 1.73 | 5.32 | <0.1 | <0.1 | <0.1 | <0.1 |
| C11 | 0.17 | 0.26 | <0.1 | <0.1 | 4.24 | 0.89 |
| C12 | 0.12 | 0.12 | <0.1 | <0.1 | <0.1 | <0.1 |
| C13 | <0.1 | <0.1 | <0.1 | <0.1 | <0.1 | <0.1 |
| C14 | <0.1 | <0.1 | <0.1 | <0.1 | <0.1 | <0.1 |
| C15 | <0.1 | <0.1 | <0.1 | <0.1 | <0.1 | <0.1 |
| C16 | <0.1 | 0.61 | <0.1 | <0.1 | 1.34 | 0.86 |
| C17 | 15.9 | 7.53 | <0.1 | 0.18 | 1.83 | 1.03 |
| C18 | 0.1 | <0.1 | <0.1 | <0.1 | <0.1 | <0.1 |
| C19 | 0.55 | 0.76 | <0.1 | <0.1 | <0.1 | <0.1 |
| C20 | <0.1 | <0.1 | <0.1 | <0.1 | <0.1 | <0.1 |
| C21 | <0.1 | <0.1 | <0.1 | <0.1 | <0.1 | <0.1 |
| C22 | <0.1 | <0.1 | <0.1 | <0.1 | <0.1 | <0.1 |
| C23 | <0.1 | <0.1 | <0.1 | <0.1 | <0.1 | <0.1 |
| C24 | <0.1 | <0.1 | <0.1 | <0.1 | <0.1 | <0.1 |
| C25 | <0.1 | <0.1 | <0.1 | <0.1 | <0.1 | <0.1 |
| C26 | <0.1 | <0.1 | <0.1 | <0.1 | <0.1 | <0.1 |
| C27 | <0.1 | 0.2 | <0.1 | <0.1 | <0.1 | <0.1 |
| C28 | <0.1 | <0.1 | <0.1 | <0.1 | <0.1 | <0.1 |
| C29 | <0.1 | <0.1 | <0.1 | <0.1 | 0.2 | <0.1 |
| C30 | <0.1 | <0.1 | <0.1 | <0.1 | <0.1 | <0.1 |
| C31 | <0.1 | <0.1 | <0.1 | <0.1 | <0.1 | <0.1 |
| C32 | 0.32 | 3.56 | <0.1 | <0.1 | <0.1 | <0.1 |
| C33 | 0.16 | 0.20 | <0.1 | <0.1 | <0.1 | <0.1 |
| C34 | <0.1 | 0.35 | <0.1 | <0.1 | 0.43 | <0.1 |
| C35 | <0.1 | 0.42 | <0.1 | <0.1 | <0.1 | <0.1 |
| C36 | <0.1 | <0.1 | <0.1 | <0.1 | <0.1 | <0.1 |
| C37 | <0.1 | <0.1 | <0.1 | <0.1 | <0.1 | <0.1 |
| C38 | <0.1 | <0.1 | <0.1 | <0.1 | <0.1 | <0.1 |
| C39 | <0.1 | <0.1 | <0.1 | <0.1 | <0.1 | <0.1 |
| C40 | <0.1 | <0.1 | <0.1 | <0.1 | <0.1 | <0.1 |

1. bee venom sIgE, ImmunoCAP
2. wasp venom sIgE, ImmunoCAP
3. IgE-reactivity to non-glycosylated rApi m 1 measured by allergen micro-array
4. IgE-reactivity to non-glycosylated rApi m 1 measured by ImmunoCAP
5. IgE-reactivity to rVes v 5 measured by allergen micro-array
6. IgE-reactivity to rVes v 5 measured by ImmunoCAP

Abbr: IgE-levels ≥ 0.1 ISU, ≥ 0.35 kU_A_/L are highlighted in grey
