# Supplementary material for: The culprit insect but not severity of allergic reactions to bee and wasp venom can be determined by molecular diagnosis
Source: PLoS One. 2018 Jun 25;13(6):e0199250. doi: 10.1371/journal.pone.0199250 (PMC6016944; doi:10.1371/journal.pone.0199250)
Supplement: S3 Table — (DOCX) [file pone.0199250.s007.docx]

**S3 Table. IgE–reactivity to marker allergens for carbohydrate sensitization and to the major timothy grass pollen allergens in the German population**

| Pat  no. | nPhl p 4^a)^ | nCyn d 1^b)^ | nCup a 1^c)^ | nJug r 2^d)^ | HRP^e)^ | MUXF3^f)^ | ASOD^g)^ | rPhl p 1^h)^ | rPhl p 5^h)^ | rPhl p 6^h)^ |
| --- | --- | --- | --- | --- | --- | --- | --- | --- | --- | --- |
|  | ISU | ISU | ISU | ISU | kU_A_/L | kU_A_/L | kU_A_/L | ISU | ISU | ISU |
| G1 | 6.78 | 8.75 | <0.1 | 0.2 | n.d. | n.d. | n.d. | 48.85 | 10.55 | 11.11 |
| G2 | 1.68 | 2.04 | <0.1 | 0.38 | n.d. | n.d. | n.d. | <0.1 | <0.1 | <0.1 |
| G3 | 0.41 | 0.38 | <0.1 | <0.1 | n.d. | n.d. | n.d. | 0.97 | <0.1 | <0.1 |
| G4 | 2.41 | 1.34 | 1.4 | 0.89 | 41.6 | 3.23 | 9.94 | <0.1 | <0.1 | <0.1 |
| G5 | 6.8 | 1.19 | <0.1 | 0.24 | 47.1 | 11.1 | 11.6 | 9.29 | <0.1 | <0.1 |
| G6 | 4.51 | 4.33 | 6.46 | 1.47 | n.d. | n.d. | n.d. | <0.1 | <0.1 | <0.1 |
| G7 | 2.45 | 2.88 | <0.1 | 0.82 | 6.2 | <0.1 | 1.3 | <0.1 | <0.1 | <0.1 |
| G8 | 0.4 | 0.18 | 0.24 | <0.1 | n.d. | n.d. | n.d. | <0.1 | <0.1 | <0.1 |
| G9 | 7.77 | 7.44 | 1.17 | 1.38 | n.d. | n.d. | n.d. | 22.2 | 10.12 | 1.71 |
| G10 | 2.77 | 2.11 | 0.56 | <0.1 | n.d. | n.d. | n.d. | <0.1 | <0.1 | <0.1 |
| G11 | <0.1 | <0.1 | <0.1 | <0.1 | n.d. | n.d. | n.d. | <0.1 | <0.1 | <0.1 |
| G12 | <0.1 | <0.1 | <0.1 | <0.1 | n.d. | n.d. | n.d. | <0.1 | <0.1 | <0.1 |
| G13 | 14.53 | 6.55 | <0.1 | 0.98 | n.d. | n.d. | n.d. | 16.72 | 10.12 | 1.72 |
| G14 | 18.65 | 17.18 | 0.57 | 3.5 | 21.9 | 7.22 | 12.9 | 2.39 | <0.1 | <0.1 |
| G15 | 9.34 | 9.34 | 4.25 | 4.3 | 53.3 | 13.7 | 13.2 | <0.1 | <0.1 | <0.1 |
| G16 | <0.1 | <0.1 | <0.1 | <0.1 | 0.14 | <0.1 | <0.1 | <0.1 | <0.1 | <0.1 |
| G17 | <0.1 | <0.1 | <0.1 | <0.1 | n.d. | n.d. | n.d. | <0.1 | <0.1 | <0.1 |
| G18 | <0.1 | <0.1 | <0.1 | <0.1 | n.d. | n.d. | n.d. | 1.23 | 6.68 | <0.1 |
| G19 | 2.57 | 0.64 | 1.52 | 2.57 | 26.7 | 5.79 | 14.6 | <0.1 | <0.1 | <0.1 |
| G20 | 0.34 | 0.27 | 0.19 | <0.1 | 1 | <0.1 | 0.3 | <0.1 | <0.1 | <0.1 |
| G21 | 0.21 | 0.23 | <0.1 | <0.1 | 10.9 | 1.08 | 1.74 | <0.1 | <0.1 | <0.1 |
| G22 | 0.61 | 0.62 | <0.1 | 0.3 | 1.35 | 0.4 | 0.76 | <0.1 | <0.1 | <0.1 |
| G23 | 0.97 | 1.15 | 0.43 | 0.37 | 19.6 | 1.31 | 4.14 | <0.1 | <0.1 | <0.1 |
| G24 | 0.78 | 0.77 | 0.58 | 0.22 | 3.14 | <0.1 | 0.57 | <0.1 | <0.1 | <0.1 |
| G25 | 13.44 | 11.5 | 0.31 | 2.96 | 49.9 | 18.6 | 37.3 | 1.01 | <0.1 | <0.1 |
| G26 | 1.95 | 1.92 | 0.73 | 0.55 | n.d. | n.d. | n.d. | <0.1 | <0.1 | <0.1 |
| G27 | <0.1 | <0.1 | <0.1 | <0.1 | <0.1 | 0.15 | 0.24 | <0.1 | <0.1 | <0.1 |
| G28 | 1.05 | 1.22 | 0.68 | 0.33 | 5.73 | 0.38 | 2.4 | <0.1 | <0.1 | <0.1 |
| G29 | <0.1 | <0.1 | <0.1 | <0.1 | <0.1 | <0.1 | <0.1 | <0.1 | <0.1 | <0.1 |
| G30 | 1.02 | 4.4 | 0.46 | <0.1 | 4.93 | 0.46 | 2.32 | 10.4 | 2.27 | <0.1 |
| G31 | <0.1 | <0.1 | <0.1 | <0.1 | n.d. | n.d. | n.d. | <0.1 | <0.1 | <0.1 |
| G32 | <0.1 | <0.1 | <0.1 | <0.1 | n.d. | n.d. | n.d. | <0.1 | <0.1 | <0.1 |
| G33 | <0.1 | <0.1 | <0.1 | <0.1 | n.d. | n.d. | n.d. | <0.1 | <0.1 | <0.1 |
| G34 | 0.39 | 0.59 | 0.38 | <0.1 | 1.78 | 0.33 | 1.62 | <0.1 | <0.1 | <0.1 |
| G35 | 0.14 | <0.1 | <0.1 | <0.1 | <0.1 | 0.15 | <0.1 | <0.1 | <0.1 | <0.1 |
| G36 | <0.1 | <0.1 | <0.1 | <0.1 | n.d. | n.d. | n.d. | <0.1 | <0.1 | <0.1 |
| G37 | 2.41 | 2.96 | 0.82 | 0.92 | 20.5 | 1.17 | 4.86 | <0.1 | <0.1 | <0.1 |
| G38 | 1.53 | 1.01 | 0.68 | 0.32 | 4.71 | 2.49 | 2.54 | <0.1 | <0.1 | <0.1 |
| G39 | 1.86 | 2.7 | <0.1 | 0.3 | n.d. | n.d. | n.d. | 9.35 | 6.17 | 0.63 |
| G40 | 0.46 | 0.4 | <0.1 | <0.1 | 1.6 | 0.26 | 0.34 | <0.1 | <0.1 | <0.1 |
| G41 | 19.01 | 16.93 | 1.82 | 3.61 | 79.7 | 36.9 | 49.6 | 0.75 | <0.1 | <0.1 |
| G42 | <0.1 | <0.1 | <0.1 | <0.1 | <0.1 | <0.1 | <0.1 | <0.1 | <0.1 | <0.1 |
| G43 | 1.85 | 1.39 | <0.1 | 0.45 | 8.84 | 6.63 | 5.72 | <0.1 | <0.1 | <0.1 |
| G44 | <0.1 | <0.1 | <0.1 | <0.1 | 0.28 | <0.1 | <0.1 | <0.1 | <0.1 | <0.1 |
| G45 | 0.73 | <0.1 | <0.1 | <0.1 | 2.35 | 0.53 | 1.86 | 8.22 | 0.74 | <0.1 |
| G46 | 1.01 | 0.62 | <0.1 | 0.24 | 7.24 | 0.6 | 2.55 | <0.1 | <0.1 | <0.1 |
| G47 | <0.1 | <0.1 | <0.1 | <0.1 | 1.02 | <0.1 | 0.22 | <0.1 | <0.1 | <0.1 |
| G48 | <0.1 | <0.1 | <0.1 | <0.1 | <0.1 | <0.1 | <0.1 | <0.1 | <0.1 | <0.1 |
| G49 | 10.58 | 6.11 | <0.1 | 3.64 | 41.6 | 8.13 | 25.1 | <0.1 | <0.1 | <0.1 |
| G50 | 4.88 | 8.55 | <0.1 | <0.1 | <0.1 | <0.1 | <0.1 | 9.19 | <0.1 | <0.1 |
| G51 | 18.63 | 21.76 | 0.75 | 0.77 | 9.97 | 5.47 | 5.94 | 49.23 | 26.07 | 7.63 |
| G52 | 7.06 | 5.83 | 0.73 | 0.37 | 14.6 | 0.47 | 2.38 | 48.42 | 17.82 | <0.1 |
| G53 | <0.1 | <0.1 | <0.1 | <0.1 | <0.1 | <0.1 | <0.1 | <0.1 | <0.1 | <0.1 |
| G54 | 36.24 | 18.27 | <0.1 | <0.1 | n.d. | n.d. | n.d. | 67.43 | 58.69 | 3.33 |
| G55 | 10.22 | 9.89 | 7.33 | 1.99 | 14.8 | 0.34 | 2.57 | 0.32 | 3.99 | <0.1 |
| G56 | 0.51 | 0.41 | <0.1 | <0.1 | n.d. | n.d. | n.d. | <0.1 | <0.1 | <0.1 |
| G57 | 0.8 | 0.72 | <0.1 | <0.1 | 0.6 | 0.14 | 0.47 | 0.64 | 5.58 | 3.24 |
| G58 | 0.4 | 0.46 | 0.33 | <0.1 | n.d. | n.d. | n.d. | <0.1 | <0.1 | <0.1 |
| G59 | 0.51 | <0.1 | <0.1 | <0.1 | n.d. | n.d. | n.d. | <0.1 | <0.1 | <0.1 |
| G60 | 29.57 | 32.64 | 0.67 | 7.01 | 14.7 | 2.95 | 9.49 | <0.1 | <0.1 | <0.1 |
| G61 | 0.43 | <0.1 | <0.1 | <0.1 | n.d. | n.d. | n.d. | <0.1 | <0.1 | <0.1 |
| G62 | 0.62 | 0.53 | <0.1 | <0.1 | n.d. | n.d. | n.d. | <0.1 | <0.1 | <0.1 |
| G63 | <0.1 | <0.1 | <0.1 | <0.1 | 0.17 | <0.1 | <0.1 | <0.1 | <0.1 | <0.1 |
| G64 | 1.08 | 6.44 | <0.1 | 0.46 | n.d. | n.d. | n.d. | 34.94 | 18.38 | 4.06 |
| G65 | 0.28 | <0.1 | <0.1 | <0.1 | 0.25 | <0.1 | <0.1 | <0.1 | <0.1 | <0.1 |
| G66 | 6.05 | 5.2 | <0.1 | 1.46 | n.d. | n.d. | n.d. | <0.1 | <0.1 | <0.1 |
| G67 | 6.15 | 10.84 | 2.39 | 1.99 | n.d. | n.d. | n.d. | 13.39 | 14.68 | 3.68 |

1. IgE-reactivity to nPhl p 4, a glycosylated timothy grass pollen allergen
2. IgE-reactivity to nCyn d 1, a glycosylated Bermuda grass pollen allergen
3. IgE-reactivity to nCup a 1, a glycosylated cypress pollen allergen
4. IgE-reactivity to nJug r 2, a glycosylated walnut allergen
5. IgE-reactivity to horseradish peroxidase
6. IgE-reactivity to glycosylated Bromelain
7. IgE-reactivity to ascorbate oxidase
8. IgE-reactivity to rPhl p 1, rPhl p 5 or rPhl p 6, marker allergens for grass pollen sensitization

Abbr: n.d.: not done

IgE-levels ≥ 0.1 ISU (micro-array), ≥ 0.35 kU_A_/L (ImmunoCAP) are highlighted in grey
