# Supplementary material for: The culprit insect but not severity of allergic reactions to bee and wasp venom can be determined by molecular diagnosis
Source: PLoS One. 2018 Jun 25;13(6):e0199250. doi: 10.1371/journal.pone.0199250 (PMC6016944; doi:10.1371/journal.pone.0199250)
Supplement: S4 Table — (DOCX) [file pone.0199250.s008.docx]

**S4 Table. IgE-reactivity to marker allergens for carbohydrate sensitization and to the major timothy grass pollen allergens in mono-sensitized Slovenian population**

| Pat  no. | nPhl p 4 ^a)^ | nCyn d 1 ^b)^ | nCup a 1 ^c)^ | nJug r 2 ^d)^ | HRP ^e)^ | MUXF3 ^f)^ | ASOD ^g)^ | rPhl p 1 ^h)^ | rPhl p 5 ^h)^ | rPhl p 6 ^h)^ |
| --- | --- | --- | --- | --- | --- | --- | --- | --- | --- | --- |
|  | ISU | ISU | ISU | ISU | kU_A_/L | kU_A_/L | kU_A_/L | ISU | ISU | ISU |
| S1 | <0.1 | <0.1 | <0.1 | <0.1 | n.d. | n.d. | n.d. | <0.1 | <0.1 | <0.1 |
| S2 | <0.1 | <0.1 | <0.1 | <0.1 | n.d. | n.d. | n.d. | <0.1 | <0.1 | <0.1 |
| S3 | 0.68 | 0.25 | <0.1 | 0.25 | n.d. | n.d. | n.d. | <0.1 | <0.1 | <0.1 |
| S4 | <0.1 | <0.1 | <0.1 | <0.1 | n.d. | n.d. | n.d. | <0.1 | <0.1 | <0.1 |
| S5 | <0.1 | <0.1 | <0.1 | <0.1 | n.d. | n.d. | n.d. | <0.1 | <0.1 | <0.1 |
| S6 | <0.1 | <0.1 | <0.1 | <0.1 | n.d. | n.d. | n.d. | <0.1 | <0.1 | <0.1 |
| S7 | 0.26 | 0.21 | 0.16 | 0.22 | n.d. | n.d. | n.d. | <0.1 | <0.1 | <0.1 |
| S8 | <0.1 | <0.1 | <0.1 | <0.1 | n.d. | n.d. | n.d. | <0.1 | <0.1 | <0.1 |
| S9 | <0.1 | <0.1 | <0.1 | <0.1 | n.d. | n.d. | n.d. | <0.1 | <0.1 | <0.1 |
| S10 | <0.1 | <0.1 | <0.1 | <0.1 | n.d. | n.d. | n.d. | <0.1 | <0.1 | <0.1 |
| S11 | 9.43 | 6.94 | <0.1 | 1.31 | n.d. | n.d. | n.d. | <0.1 | <0.1 | <0.1 |
| S12 | <0.1 | <0.1 | <0.1 | <0.1 | n.d. | n.d. | n.d. | <0.1 | <0.1 | <0.1 |
| S13 | <0.1 | <0.1 | <0.1 | <0.1 | n.d. | n.d. | n.d. | <0.1 | <0.1 | <0.1 |
| S14 | <0.1 | <0.1 | <0.1 | <0.1 | n.d. | n.d. | n.d. | <0.1 | <0.1 | <0.1 |
| S15 | <0.1 | <0.1 | <0.1 | <0.1 | n.d. | n.d. | n.d. | <0.1 | <0.1 | <0.1 |
| S16 | 1.29 | 1.2 | 0.51 | 0.25 | n.d. | n.d. | n.d. | <0.1 | <0.1 | <0.1 |
| S17 | <0.1 | <0.1 | <0.1 | <0.1 | n.d. | n.d. | n.d. | <0.1 | <0.1 | <0.1 |
| S18 | 0.22 | <0.1 | <0.1 | <0.1 | n.d. | n.d. | n.d. | 0.11 | <0.1 | <0.1 |
| S19 | <0.1 | <0.1 | <0.1 | <0.1 | n.d. | n.d. | n.d. | <0.1 | <0.1 | <0.1 |
| S20 | <0.1 | <0.1 | <0.1 | <0.1 | n.d. | n.d. | n.d. | 0.16 | <0.1 | <0.1 |

1. IgE-reactivity to nPhl p 4, a glycosylated timothy grass pollen allergen
2. IgE-reactivity to nCyn d 1, a glycosylated Bermuda grass pollen allergen
3. IgE-reactivity to nCup a 1, a glycosylated cypress pollen allergen
4. IgE-reactivity to nJug r 2, a glycosylated walnut allergen
5. IgE-reactivity to horseradish peroxidase
6. IgE-reactivity to glycosylated Bromelain
7. IgE-reactivity to ascorbate oxidase
8. IgE-reactivity to rPhl p 1, rPhl p 5 or rPhl p 6, marker allergens for grass pollen sensitization

Abbr: n.d.: not done

IgE-levels ≥ 0.1 ISU (micro-array), ≥ 0.35 kU_A_/L (ImmunoCAP) are highlighted in grey
